# Safety studies with the oral rabies virus vaccine strain SPBN GASGAS in the small Indian mongoose (*Herpestes auropunctatus*)

**DOI:** 10.1186/s12917-018-1417-0

**Published:** 2018-03-13

**Authors:** Steffen Ortmann, Ad Vos, Antje Kretzschmar, Nomusa Walther, Christiane Kaiser, Conrad Freuling, Ivana Lojkic, Thomas Müller

**Affiliations:** 1 IDT Biologika GmbH, Am Pharmapark, 06861 Dessau Rosslau, Germany; 2 Institute of Molecular Virology and Cell Biology, Friedrich-Loeffler-Institute, Südufer 10, 17493 Greifswald, Insel Riems Germany; 3 Department of Virology, Croatian Veterinary Institute, Svaská cesta 143, 10000 Zagreb, Croatia

**Keywords:** Rabies, Mongoose, Oral vaccination, Safety, SPBN GASGAS

## Abstract

**Background:**

Oral vaccination of the small Indian mongoose against rabies has been suggested as a potential tool to eliminate mongoose-mediated rabies on several Caribbean islands. A recently developed oral rabies virus vaccine strain, SPBN GASGAS, has already been shown to be efficacious in this reservoir species. Since, all available oral rabies vaccines are based on replication-competent viruses and vaccine baits are distributed unsupervised in the environment, enhanced safety standards for such vaccine types are required.

**Results:**

The results of safety studies, including overdose, repeated doses, dissemination and different routes of administration, in the target species are presented. It was shown that the construct was apathogenic, irrespective of dose and route of administration. Even when it was inoculated directly in the brain, it did not induce rabies infection. Furthermore, the vaccine strain did not spread within the target species after direct oral instillation beyond the site of entry.

**Conclusion:**

The vaccine strain SPBN GASGAS meets the safety requirements for live rabies virus vaccines in this target species, the small Indian mongoose.

## Background

The small Indian mongoose (*Herpestes auropunctatus*), native to southern Asia, has been introduced to many islands worldwide for control of pest animals [1]. Since the mid-twentieth century, the species has been identified as a rabies reservoir on some of the Caribbean islands it has been introduced to, i.e. Cuba, Dominican Republic, Grenada and Puerto Rico [2]. As with other wildlife rabies reservoirs, attempts to control mongoose rabies in the Caribbean through culling have so far failed [1, 2]. Hence, oral vaccination of mongoose against rabies has been suggested as a more promising alternative [3, 4] and is presently under investigation in Puerto Rico [5]. Oral vaccination campaigns against rabies of different wildlife reservoir species has been successfully implemented in Europe and North America [6, 7]. Several experimental studies have shown that mongoose can be vaccinated against rabies by the oral route using the vaccine strain SPBN GASGAS [4, 8]. Also, field studies showed that baits were readily accepted by mongooses and can reach a high proportion of the target species [9–11]. Thus oral vaccination of small Indian mongooses against rabies seems a feasible approach as all three components of this concept (vaccine, bait and distribution system) are available.

However, besides being efficacious after a single dose, the vaccine should also meet certain safety standards; especially considering that all available oral rabies vaccines are based on replication-competent viruses. Furthermore, in contrast to parenteral administered vaccines, oral rabies vaccine baits are distributed in the environment without direct supervision. Therefore, an important prerequisite for the deliberate distribution of vaccine baits in the environment containing live replication competent viruses is a stringent safety assessment. In this paper, the safety studies in the potential target species, the small Indian mongoose, with the oral rabies virus vaccine strain SPBN GASGAS are presented. For the licensing of such vaccines in Puerto Rico, the Center of Veterinary Biologics of the Animal and Plant Health Inspection Service, United States Department of Agriculture (USDA/APHIS/CVB), is responsible. Detailed descriptions of the safety studies required can be found in the Code of Federal Regulation (Title 9 – Animals and Animal Products) [12] and Veterinary Services Memoranda [13]. However, unlike in the European Pharmacopoeia there is no dedicated chapter on oral rabies vaccines in these documents; the listed study protocols for live rabies virus vaccines are intended for parenteral administered vaccines. Thus, it became necessary to adapt the protocols in such a way that the recommended oral route of administration was investigated instead of intramuscularly injection. Besides the required dissemination study of the vaccine virus using the recommended route of administration (oral), animals also received the vaccine virus by the i.c. and i.m route. Repeated dose studies are required only for vaccines that need to be administered on multiple occasions in order to induce a protective immune response. For oral vaccination of mongoose against rabies a single dose SPBN GASGAS is sufficient [4, 8]. Hence, a repeated dose study is not compulsory. However, in the context of oral vaccination where any animal can locate and consume multiple vaccine baits distributed in the environment repeated dose studies are relevant for safety assessment; thus, a single high-titred overdose was administered. Furthermore, mongooses can come in contact with the vaccine virus on multiple occasions after bait distribution. To imitate such a situation, the animals were offered a vaccine dose on three occasions within 1 week. The selection of 7 days is based on the observation that most baits distributed in the environment are generally taken up within this time frame [14–17]. Also, stability of SPBN GASGAS over periods longer than 7 days in the environment is not guaranteed, especially considering the prevailing climatic conditions in Puerto Rico. Rabies lyssavirus is considered relatively thermo-instable and will rapidly lose its infectivity when exposed to elevated temperatures [18]. Shedding of vaccine virus in saliva and feces has been investigated during the dissemination and overdose studies; these results have been published previously and will not be presented here [19]. It was shown that the vaccine strain SPBN GASGAS was completely innocuous for the small Indian mongoose, irrespective of the route of administration and dose.

## Methods

### Animals

Male juvenile and adult mongooses were caught using baited box traps on the rabies-free island Korčula, Croatia, and transported to IDT Biologika GmbH (IDT), Germany. Each mongoose was housed in a cage individually (0.44 m^2^ - including sleeping box) and identified by cage number. The cages were placed within the two rooms of an isolation unit. Temperature in the isolation unit was between 20 °C and 25 °C, humidity between 20% and 70%, the light regimen was 12 h light and 12 h darkness and the air exchange rate was 15 times per hour. The mongooses were fed with a standardised feed of the following composition throughout the study: 26% poultry, 22% fish scraps, 12% beef heart, 5% beef liver/beef kidney, 12% green tripe, 16% grain premix for fur animals with vitamins, minerals and water (Schirmer und Partner, 09306 Döhlen, Germany). Every animal received 35 – 50 g feed per day according to individual consumption behaviour and need. Additionally, twice a week, the mongooses received some fruit or vegetables and a boiled egg. Water was offered ad libitum. Animals were checked at least once per day and the individual general health status, food intake and defecation was recorded. In case the mongoose needed to be sedated or euthanized the following capture procedure was followed; a small cage-trap with a fixation mechanism was placed in front of the sleeping box. Once the animals were trapped, they received an intramuscular injection into the thigh muscles of a hind leg with a solution of Ketamin (26.5 mg/kg; Ketamin 10%, MEDISTAR Arzneimittelvertrieb GmbH, Germany) and Xylazin (0.9 mg/kg; Sedaxylan®, Eurovet Animal Health B.V.; Netherland). The euthanasia at the end of the studies was done under deep sedation by an intracardial injection with Pentorbital-Natrium (450 mg/kg; Release® 500 mg/ml; Wirtschaftsgenossenschaft deutscher Tierärzte eG; Germany).

### Vaccine

The vaccine strain SPBN GASGAS is derived from SAD L16, a cDNA clone of the oral rabies virus vaccine strain SAD B19 [19, 20]. SPBN GASGAS lacks the pseudogene (Ψ) and has been genetically modified by site-directed mutagenesis at the amino acid positions 194 and 333 of the glycoprotein [21]. Furthermore, the construct contains a second identical glycoprotein gene with the described modifications [22, 23]. Two different doses, 10^7.7^ and 10^9.1^ FFU/ml SPBN GASGAS, were used in the studies. The virus material was produced according to the protocol given by Vos et al. [24]. Material for the overdose study was concentrated via tangential flow filtration using ultrafiltration flat sheet cassettes with a Molecular Weight Cut Off (MWCO) of 300 kDa.

### Study protocol

As mentioned, there is no dedicated monograph from the regulatory authorities for oral rabies vaccines available in the US. Therefore, small adaptations have been made to the existing guidelines that are intended for live rabies virus vaccines delivered parenterally; 9 CFR 113.312 “Rabies Vaccine, Live Virus” (Table 1). The number of animals per study was based on the minimum numbers required by the regulatory authorities; to compensate for potential losses for the long observation period in the overdose study several surplus animals were included.

**Table 1:** Overview of safety studies conducted in the small Indian mongoose

Purpose	Number of animals	Observation period (days)	Dose (titre; volume)
Repeated dose (day 0, 3 & 7)	5	56	10^7.7^ FFU/ml; 0.7 ml
Dissemination	12	1, 2, 3, 5, 7 & 10	10^7.7^ FFU/ml; 0.7 ml
Overdose	14	182	10^9.1^ FFU/ml; 0.7 ml
Different routes of administration	5 (i.m.)	15, 20, 25, 30 & 35	10^7.7^ FFU/ml; 1.0 ml
	5 (d.o.a)	15, 20, 25, 30 & 35	10^7.7^ FFU/ml; 1.0 ml
	5 (i.c.)	3, 6, 9, 15 & 30	10^7.7^ FFU/ml; 0.1 ml

#### Overdose

According to 9 CFR 113.312, it is required to administer 10 doses of high virus titer material i.m. and by infiltrating a major nerve of at least 10 animals each and observe them for 90 days. As the intended route of administration is by offering the animals a vaccine bait the protocol has been adapted and 14 animals received a single dose (0.7 ml) of high-titred SPBN GASGAS (10^9.1^ FFU/ml) by direct oral application onto the mucosae of the *palatum durum*, cheeks and under the tongue. Subsequently, the animals were observed for 182 days post administration. Blood samples were collected on day 0, 28 and 182 post vaccine administration and examined for the presence of virus neutralizing antibodies (VNA). Finally, the brain of the animals was examined for the presence of rabies virus antigen at the end of the observation period.

#### Repeated dose

5 mongooses received a dose of 0.7 ml of the vaccine strain (10^7.7^ FFU/ml) by direct oral application on study days 0, 3 and 7. The observation period ended 56 days after the first administration of the vaccine strain and all animals were euthanized. Subsequently, the brain was examined for the presence of virus antigen. Blood samples were collected from all animals on study days 0, 28 and 56 for determination of the antibody titre.

#### Dissemination

For this purpose 12 mongooses received one dose (0.7 ml) SPBN GASGAS with a titre of 10^7.7^ FFU/ml. On study days 1, 2, 3, 5, 7 and 10 post administration two treated animals were euthanized and subsequently organs and tissues were collected to test for the presence of the vaccine strain. The following tissue samples were collected of all animals from the oral cavity; regional lymphnodes (*lnn. Mandibularis, lnn. reteropharyngeales*), upper and lower mucosa, tongue, soft palate, tonsils (*tonsilla sublingualis, tonsilla lingualis, tonsilla palatine, tonsilla pharyngica*). Additionally, samples from the brain (*hippocampus, medulla oblongata, cerebrum* and *cerebellum*) and additional tissues from the lungs, kidneys, bladder, heart, colon, jejunum and ileum were collected. Blood samples were collected prior to vaccination and at the time point of euthanasia for determination of seroconversion.

#### Different routes of administration

According to 9 CFR 113.312, 5 animals each should receive the vaccine intramuscularly (i.m.) or by infiltrating a major nerve and its surrounding tissue. The selected routes of administration have been slightly adapted for the intended oral use. Besides injecting the vaccine strain i.m., it was also administered by direct oral administration (d.o.a.). The d.o.a.-treated animals received 1.0 ml (10^7.7^ FFU/ml) of the vaccine strain by direct application onto the mucosae of the pharynx and oral cavity after sedation. The other group received 1.0 ml SPBN GASGAS (10^7.7^ FFU/ml) divided over 4 injection sites; the thigh muscles of both hind limbs and in the muscles of both shoulder blades. One animal of each group (*n* = 5) was sacrificed on day 15, 20, 25, 30 and 35 post vaccine administration. Instead of infiltrating a major nerve, the vaccine strain was directly inoculated intracerebrally (i.c.) in 5 animals. These animals were put under anaesthesia and shaved along a line between the outer canthus and area of the medial cranial line. This point was disinfected with iodine solution. An incision of about 1 cm in length was performed in the cranial-caudal direction up to the cranial bone. Small blood losses were stopped by tamponade. The skin along the wound was displaced to the side, and the superimposed muscle layers freed from the cranium. A hole measuring about 0.2 cm in diameter was drilled through the skull (trepanation) using a drill (DREMEL® 8200, DREMEL Europe, Netherlands). Using a 23G cannula, an inoculum of 0.1 ml (10^7.7^ FFU/ml) was applied 1.0 cm deep into the left half of the cerebrum. The wound was closed with two individual staples and freed from clotted blood. On day 3, 6, 9, 15 and 30 post administration one animal was sacrificed. For all animals, the brain, salivary glands, and kidneys were collected and tested for the presence of the vaccine strain. In addition, the *Lnn. mandibularis* and *Lnn. retropharyngeales* were taken from the d.o.a group and in the i.m. group regional lymph nodes were additionally taken near the application site.

### Diagnostic assays

Rabies viral antigen in the brain (Ammons’ horn, cerebellum and Medulla oblongata) of animals was detected by the fluorescent antibody test (FAT) using commercial monoclonal FITC- labelled anti-rabies antibodies (SIFIN, Berlin, Germany) and defined positive - and negative controls [25]. Tissues samples (10 mg – 100 mg) other than brain were homogenized in a volume of 1000 μl sterile minimum essential medium (MEM-10, with 2% Streptomycin). Subsequently, RNA was extracted from 200 μl organ suspension using the commercial RNeasy kit (Qiagen, Germany) and screened for vaccine viral RNA using a rabies virus specific RT-qPCR essentially as described [26]. RT-qPCR positive tissue samples were subject to investigation for presence of replication competent vaccine strain in the Rabies Tissue Culture Infection Test (RTCIT) on murine neuroblastoma (Na42/13) cells [27]. Three consecutive passages were conducted to confirm a negative result. Also, in case brain samples were FAT-inconclusive, the samples were subsequently examined by RTCIT.

Sera were tested for the presence of VNA in a modified rapid fluorescence focus inhibition test (RFFIT) and the Fluorescent Antibody Viral Neutralization (FAVN) essentially as described by Moore et al [28] and Cliquet et al [29], respectively, using rabies lyssavirus CVS-11 as test virus and BHK21-BSR/5 (CCLV-RIE 0194/260) cells. While the FAVN was used in the overdose study, RFFIT was applied in the other three studies. In each assay, the calibrated WHO international standard immunoglobulin (2nd human rabies immunoglobulin preparation, National Institute for Standards and Control, Potters Bar, UK) or the calibrated OIE serum of dog origin adjusted to 0.5 international units (IU) and a naive bovine serum served as positive – and negative control, respectively. VNA titres were calculated using inverse interpolation as described and subsequently converted into concentrations expressed in international units (IU/mL) [30]. VNA titres equal or greater than 0.5 IU/ml were considered positive.

## Results

### Overdose

2 of the 14 animals were euthanized before the end of the observation period: on day 38 and 56 post vaccine administration. The clinical signs observed did not indicate rabies and no rabies virus antigen was detected by FAT in the brains of these animals. Upon necropsy, both animals showed a hepatitis of unknown origin. All other animals showed no impairment of general health throughout the study, including body weight loss, and tested negative for the presence of rabies virus antigen in the brain. All animals tested seronegative before vaccination and subsequently seroconverted, except for one animal, post vaccine administration (FAVN). The geometric mean titre (GMT) was 8.32 IU/ml [range: 0.06–30.81] and 8.36 IU/ml [range: 1.98–30.81] on day 28 and 182 post vaccine administration, respectively (Table 2).

**Table 2:** Serological results of the overdose study

Animal	B0	B1	B2
	day 0	day 28	day 182
1	0.04	7.81	2.60
2	0.04	13.52	23.42
3	0.04	30.81	n.d.
4	0.04	30.81	30.81
5	0.04	0.06	n.d.
6	0.04	17.79	23.42
7	0.04	3.43	3.43
8	0.04	1.14	3.43
9	0.04	30.81	23.42
10	0.04	13.52	1.98
11	0.04	30.81	5.93
12	0.04	23.42	23.42
13	0.04	10.27	4.51
14	0.04	5.93	7.81
GMT	0.04	8.32	8.36
s.d.	–	11.70	10.93

*n.d.* not determined since no sample available since animal was removed from study prior to the end of the observation period, *GMT* geometric mean titre (IU/ml - FAVN), *s.d.* standard deviation

### Repeated dose

All animals remained healthy and no critical clinical findings were observed during the entire observation period of 56 days. The brains of all study animals tested negative for the presence of rabies virus antigen (FAT). All five animals tested seronegative prior to vaccine administration and all animals developed a detectable immune response after vaccine administration. The GMT was 4.39 IU/ml [range: 1.61–11.56) and 3.51 IU/ml [range: 2.33–5.66] on day 28 and 56 after the first dose was administered, respectively (Table 3).

**Table 3:** Level of virus neutralizing antibodies in sera after repeated administration of SPBN GASGAS

Animal	B0	B1	B2
	(day 0)	(day 28)	(day 56)
1	0.06	4.77	3.02
2	0.04	1.61	5.66
3	0.08	11.56	4.56
4	0.07	3.75	2.97
5	0.04	4.78	2.33
GMT	0.06	4.37	3.52
s.d.	0.02	3.73	1.37

*day* number of days after first dose administered,*GMT* geometric mean titre (IU/ml – RFFIT), *s.d.* standard deviation

### Dissemination

All but two of the selected tissues samples collected after d.o.a tested PCR-negative. While rabies virus could be isolated in RTCIT from a PCR-positive lung sample taken from an animal 1 day after oral administration, virus isolation of a PCR-positive *Tonsilla sublingualis* of another animal sampled on day 7 after application of the test material failed. Furthermore, all brain samples tested negative for the presence of rabies virus antigen. Seroconversion (> 0.5 IU/ml) was observed only in animals sacrificed 10 days after vaccine administration (Table 4).

**Table 4:** Serological results of the dissemination study: RFFIT – IU/ml

sample day (post vacc.)	Animal											
	1	2	3	4	5	6	7	8	9	10	11	12
0	0.02	0.05	0.11	0.02	0.02	0.11	0.07	0.02	0.03	0.10	0.02	0.12
1	0.08	0.09										
2			0.08	0.08								
3					0.06	0.07						
5							0.04	0.19				
7									0.42	0.16		
10											5.43	10.03

### Different routes of administration

All animals tested seronegative (< 0.5 IU/ml) prior to vaccine administration; GMT = 0.07 IU/ml, [range: 0.02–0.36 IU/ml]. During the observation period, none of the animals showed any signs of illness or died during this period, including the animals that received the vaccine strain i.c. With the exception of one lymph node sample from the hind leg collected on day 15 post vaccine administration (i.m.), all tissue samples tested negative by PCR. The positive lymph node sample was found negative for the presence of viable virus by RTCIT. No virus antigen was detected in the brain of all 5 animals; already 3 days post vaccine administration i.c. As the vaccine strain was directly inoculated in the brain, it can be concluded that it must have been rapidly cleared from the brain.

## Discussion

The native range of the small Indian mongoose stretches from the Middle East to Myanmar [31]. It has also been introduced to many islands in the Pacific and Indian Oceans, the Caribbean and Adriatic seas and to continental South America and Europe; predominantly in the late 19th and early twentieth century [1, 31]. In the Caribbean, the small Indian mongoose is present on 33 islands [1], but only on 4 islands rabies in mongoose has been reported; Grenada, Dominican Republic, Cuba and Puerto Rico [2]. The first major outbreak attributed to the small Indian mongoose started in 1950 in Puerto Rico [32]. In its natural distribution area it has not been identified as an important vector of rabies. Also elsewhere, only sporadic cases of rabies in other mongoose species are reported; for example India, Sri Lanka, Israel, and Nigeria [33–36]. The only other region where rabies in mongooses has been well described and is regularly reported is southern Africa [37–39]. In order to eliminate mongoose rabies in the Caribbean population control measures have not been successful [1, 2] and hence alternative methods have been suggested [4, 10, 40, 41]. Since several years, Wildlife Services of the Animal and Plant Health Inspection Service, United States Department of Agriculture (USDA/APHIS/WS) is investigating the possibility of oral vaccination of mongoose against rabies in Puerto Rico [5]. One of the prerequisites for oral vaccination is a highly efficacious and safe vaccine for the target species. The results of the safety studies presented here showed that this vaccine strain SPBN GASGAS is innocuous for the target species, the small Indian mongoose, irrespective of dose and route administered. Even direct inoculation in the brain did not result in rabies infection. Also, repeated application of the construct did not induce any adverse reaction in the animals. After oral administration, the vaccine did not disseminate to any of the organs or tissues examined. Only in one lung sample taken 24 h after oral administration, the vaccine virus was re-isolated. The position of the lungs and the time of sampling in relation to time of application lead to the assumption that the vaccine was most likely aspirated into the lungs during forced administration under sedation. It is highly unlikely that the vaccine would enter the respiratory tract under natural condition when an animal consumes a bait. Interestingly, no virus was found in the palatine tonsils as this was determined as the primary site of uptake after oral administration [42]. Finally, no infectious vaccine virus was detected in fecal samples and saliva swabs collected during the overdose and dissemination study, although viral RNA was detected in several of these samples [19]. All mongooses of which blood samples were collected seroconverted with the exception of one animal (overdose study), indicating successful vaccine uptake (Tables 2-4). Outside the scope of the studies presented here, additional safety studies have to be performed in non-target species, since all species inhabiting an area where baits are distributed can locate and come in contact with the vaccine virus. Hence, a risk assessment has to be carried out identifying possible non-target species for which additional safety studies need to be conducted.

## Conclusion

It can be concluded that the vaccine strain SPBN GASGAS fulfils the safety requirements for live rabies virus vaccines. Hence, this efficacious vaccine construct is a suitable candidate for oral vaccination of the small Indian mongoose against rabies.

## References

[1] Barun A, Hanson CC, Campbell KJ, Dl S, Veitch CR, Clout MN, Towns DR (2011). A review of the small Indian mongoose management and eradications on islands. Islands invasives: eradication and management.

[2] Everard COR, Everard JD (1992). Mongoose rabies in the Caribbean. Ann N Y Acad Sci.

[3] Everard COR, Baer GM, Alls ME, Moore SA (1981). Rabies serum neutralizing antibody in mongooses from Grenada. Trans R Soc Trop Med Hyg.

[4] Blanton JD, Meadows A, Murphy SM, Manangan J, Hanlon CA, Faber ML, Dietzschold B, Rupprecht CE (2006). Vaccination of small Asian mongoose (Herpestes javanicus) against rabies. J Wildl Dis.

[5] Berentsen AR, Johnson SR, VerCauteren KC, Gruver K, Boyd F, Gilbert AT, Chipman RB. Mongoose research by USDA wildlife Services in Puerto Rico, 2011 – 2015: implications for oral rabies vaccination. In: Abstracts RITA 2015, rabies in the Americas, Fort Collins, Colorado. p. 2015.

[6] Müller T, Freuling CM, Wysocki P, Roumiantzeff M, Freney J, Mettenleiter TC, Vos A (2015). Terrestrial rabies control in the European Union: historical achievements and challenges ahead. Vet J.

[7] Rosatte R (2011). Evolution of wildlife rabies control tactics. Adv Virus Res.

[8] Vos A, Kretzschmar A, Ortmann S, Lojkic I, Habla C, Müller T, Kaiser C, Hundt B, Schuster P (2013). Oral vaccination of captive small Indian mongoose against rabies. J Wildl Dis.

[9] Linhart SB, Creekmore TE, Corn JL, Whitney MD, Snyder BD, Nettles VF (1993). Evaluation of baits for oral rabies vaccination of mongooses: pilot field trials in Antigua, West Indies. J Wildl Dis.

[10] Creekmore TE, Linhart SB, Corn JL, Whitney MD, Snyder BD, Nettles VF (1994). Field evaluation of baits and baiting strategies for delivering oral vaccine to mongooses in Antigua, West Indies. J Wildl Dis.

[11] Berentsen A, Johnson SR, VerCauteren KC (2014). Bait matrix flavor preference by mongoose (Herpestes auropunctatus) in Puerto Rico: implications for oral rabies vaccination. Caribb J Sci.

[12] 9 CFR 113.312 “Rabies Vaccine, Live Virus”. https://www.gpo.gov/fdsys/pkg/CFR-2012-title9-vol1/pdf/CFR-2012-title9-vol1-sec113-312.pdf. Accessed 15 March 2016

[13] United States Department of Agriculture, Animal and Plant Health Inspection Service. Veterinary Services Memoranda. https://www.aphis.usda.gov/aphis/ourfocus/animalhealth/veterinary-biologics/biologics-regulations-and-guidance/ct_vb_vs_memos. Accessed 15 March 2016.

[14] Hadidian J, Jenkins SR, Johnston DH, Savarie PJ, Nettles VF, Manski D, Baer GM (1989). Acceptance of simulated oral rabies vaccine baits by urban raccoons. J Wildl Dis.

[15] Hable CP, Hamir AN, Snyder DE, Joyner R, French J, Nettles V, Hanlon C, Rupprecht CE (1992). Prerequisites for oral immunization of free-ranging raccoons (Procyon lotor) with a recombinant rabies virus vaccine: study site ecology and bait system development. J Wildl Dis.

[16] Blackwell BF, Seamans TW, White RJ, Patton ZJ, Bush RM, Cepek JD (2004). Exposure time of oral rabies vaccine baits relative to baiting density and raccoon population density. J Wildl Dis.

[17] Vos A, Sutor A, Selhorst T, Schwarz S, Pötzsch C, Staubach C, Müller T (2004). The spatial and temporal disappearance of different oral rabies vaccine baits. Berl Munch Tierarztl Wochenschr.

[18] Vos A, Neubert A (2002). Thermo-stability of the oral rabies virus vaccines SAD B19 and SAD P5/88. Dtsch Tierarztl Wochenschr.

[19] Vos A, Freuling C, Ortmann S, Kretzschmar A, Mayer D, Schliephake A, Müller T (2018). An assessment of shedding with the oral rabies virus vaccine strain SPBN GASGAS in target and non-target species. Vaccine.

[20] Schnell MJ, Mebatsion T, Conzelmann KK (1994). Infectious rabies viruses from cloned cDNA. EMBO J.

[21] Faber M, Faber ML, Papaneri A, Bette M, Weihe E, Dietzschold B, Schnell MJ (2005). A single amino acid change in rabies virus glycoprotein increases virus spread and enhances virus pathogenicity. J Virol.

[22] Faber M, Pulmanausahakul R, Hodawadekar SS, Spitsin S, McGettigan JP, Schnell MJ (2002). Overexpression of the rabies virus glycoprotein results in enhancement of apoptosis and antiviral immune response. J Virol.

[23] Faber M, Faber ML, Li J, Preuss MA, Schnell MJ, Dietzschold B (2007). Dominance of a nonpathogenic glycoprotein gene over a pathogenic glycoprotein gene in rabies virus. J Virol.

[24] Vos A, Neumann G, Hundt B, Neubert A, Rupprecht C, Nagarajan T (2015). Attenuated vaccines for veterinary use. Current laboratory techniques in rabies diagnosis, research and prevention, volume 1.

[25] Dean DJ, Abelseth MK, Atanasiu P, Meslin F-X, Kaplan MM, Koprowski H (1996). The fluorescent antibody test. Laboratory techniques in rabies.

[26] Hoffmann B, Freuling CM, Wakeley PR, Rasmussen TB, Leech S, Fooks AR, Beer M, Müller T (2010). Improved safety for molecular diagnosis of classical rabies viruses by use of a TaqMan real-time reverse transcription-PCR "double check" strategy. J Clin Microbiol.

[27] Webster WA, Casey GA. Virus isolation in neuroblastoma cell culture. In: Meslin FX, Kaplan MM, Kaprowski H, editors. Laboratory techniques in rabies. 4th ed. Geneva, World Health Organization; 1996. p. 96–104.

[28] Moore S, Gilbert A, Vos A, Freuling C, Ellis C, Kliemt J, Müller T (2017). Review of rabies virus antibodies from oral vaccination as a correlate of protection against lethal infection in animals. Trop Med Infect Dis.

[29] Cliquet F, Aubert M, Sagné L (1998). Development of a fluorescent antibody virus neutralisation test (FAVN test) for the quantitation of rabies-neutralising antibody. J Immunol Methods.

[30] Müller T, Selhorst T, Burow J, Schameitat A, Vos A (2006). Cross-reactive antigenicity in orally vaccinated foxes and raccoon dogs against European bat lyssavirus type 1 and 2. Dev Biol.

[31] Barun A, Simberloff D, Tcrtkovic N, Pascal M (2011). Impact of the introduced small Indian mongoose (Herpestes auropunctatus) on abundance and activity time of the introduced ship rat (Rattus rattus) and the small mammal community on Adriatic islands, Croatia. NeoBiota.

[32] Tierkel ES, Arbona G, Rivera A, De Juan A (1952). Mongoose rabies in Puerto Rico. Public Health Rep.

[33] Mani RS, Moorkoth AP, Balasubramanian P, Devi K, Madhusudana SN (2016). Rabies following mongoose bite. Indian J Med Microbiol.

[34] Patabendige CGUA, Wimalaratne O (2003). Rabies in mongooses and domestic rats in the Southern Province of Sri Lanka. Ceylon Med J.

[35] Shimshony A (1997). Epidemiology of emerging zoonoses in Israel. Emerg Infect Dis.

[36] Atuman YJ, Adawa DAY, Okewole PA, Shamaki D, Audu SW, Mshelbwala PP, Ogunkoya AB (2014). Detection of rabies antigens in the brain tissues of jackals and mongooses and its implications on public health and conservation goals in Bauchi state Nigeria. Scientific J Vet Adv.

[37] Nel LH, Sabeta CT, von Teichman B, Jaftha JB, Rupprecht CE, Bingham J (2005). Mongoose rabies in southern Africa: a re-evaluation based on molecular epidemiology. Virus Res.

[38] Davis PL, Rambaut A, Bourhy H, Holmes EC (2007). The evolutionary dynamics of canid and mongoose rabies virus in southern Africa. Arch Virol.

[39] Van Zyl N, Markotter W, Nel LH (2010). Evolutionary history of African mongoose rabies. Virus Res.

[40] Everard CO, Everard JD. Mongoose rabies. Rev Infect Dis. 1988;10:S610-4. 10.1093/clinids/10.supplement_4.s610 3060954

[41] Zieger U, Marston DA, Sharma R, Chikweto A, Tiwari K, Sayyid M, Louison B, Goharriz H, Voller K, Breed AC, Werling D, Fooks AR, Horton DL (2014). The Phylogeography of rabies in Grenada, West Indies, and implications for control. PLoS Negl Trop Dis.

[42] Vos A, Freuling CM, Hundt B, Kaiser C, Nemitz S, Neubert A, Nolden T, Teifke JP, Te Kamp V, Ulrich R, Finke S, Müller T (2017). Oral vaccination of wildlife against rabies: differences among host species in vaccine uptake efficiency. Vaccine.

